# Characteristics of children with the best and poorest first- and second-year growth during rhGH therapy: data from 25 years of the Genentech national cooperative growth study (NCGS)

**DOI:** 10.1186/1687-9856-2013-9

**Published:** 2013-05-01

**Authors:** Paul B Kaplowitz, Dorothy I Shulman, James W Frane, Joan Jacobs, Barbara Lippe

**Affiliations:** 1 Endocrinology, Children’s National Medical Center, George Washington University School of Medicine & Health Sciences, Washington, DC, USA; 2 Pediatric Endocrinology, All Children's Hospital, All Children's Hospital, St. Petersburg, and University of South Florida, Tampa, FL, USA; 3 Biostatistics, Santa Monica, CA, USA; 4 Biostatistics, Genentech, Inc., South San Francisco, CA, USA; 5 Genentech, Inc., South San Francisco, CA, USA

**Keywords:** Growth hormone deficiency, Idiopathic short stature, Growth hormone therapy

## Abstract

**Background:**

Models assessing characteristics contributing to response to recombinant human growth hormone (rhGH) response rarely address growth extremes in both years 1 and 2 or examine how children track from year to year. Using National Cooperative Growth Study (NCGS) data, we determined characteristics contributing to responsiveness to rhGH and the pattern of change from years 1 to 2.

**Patients and methods:**

Height velocity standard deviation score (HV SDS) for 2 years for prepubertal children with idiopathic GH deficiency (IGHD) (n = 1899) and idiopathic short stature (ISS) (n = 1186) treated with similar doses for two years were computed. Group 1 = HV SDS < −1; 2 = HV SDS −1 to +1; 3 = HV SDS > +1.

**Results:**

For IGHD**,** mean age was 7.5 years and similar in all groups. Year 1 HV SDS was associated with greater body mass index (BMI) SDS, lower pre-treatment HV, baseline height SDS, greater target height SDS minus height SDS, and lower maximum stimulated GH (*P* <0.0001). Year 2, 172/271 (73%) in group 1 moved to either group 2 (n = 156) or 3 (n = 16). Year 2 HV SDS was associated with greater year 1 HV SDS (*r* = 0.045, *P* <0.0001), greater BMI SDS, taller parents and lower peak GH.

For ISS**,** year 1 HV SDS was associated with greater BMI SDS and lower pre-treatment HV (*P* ≤0.0001). 109/169 (64%) in group 1 moved to group 2 (n = 90) or group 3 (n = 19). Greater year 2 HV SDS was related to year 1 HV SDS (*r* = 0.27, *P* <0.0001).

**Conclusion:**

For IGHD, multiple characteristics contributed to best first-year response but for ISS, best first-year HV SDS was associated only with BMI SDS and inversely with pre-treatment HV. For both GHD and ISS, year 1 HV SDS was not a strong enough predictor of year 2 HV SDS to use first-year HV alone to determine GH continuation.

## Introduction

While several publications have described the factors that predict responsiveness to recombinant human growth hormone (rhGH) during therapy [1-4], the factors predicting which individual patients are likely to be either the best or worst responders during both the first and the second years of therapy have been studied in less detail. In addition, the persistence of growth patterns from year 1 to 2 and the factors that influence persistence or shift have also not been clarified. We used data from the Genentech National Cooperative Growth Study (NCGS), collected over a 25-year period, to examine the responsiveness to rhGH of boys and girls who were diagnosed with either idiopathic GH deficiency (IGHD) or idiopathic short stature (ISS). Our goals were to identify factors that predicted excellent or poor growth in both the first and second years of therapy, and to determine to what extent the individual child with either poor or excellent growth in the first year had a similar growth response during the second year of rhGH therapy.

## Methods

The voluntary NCGS registry was initiated in December 1985 to collect data on children treated with rhGH for evaluation of safety and efficacy. Data entered by clinical investigators in the United States and Canada included height, weight, etiology of short stature, parental height, peak serum GH response to stimulation testing, Tanner pubertal stages, and rhGH dose for consenting patients treated with Genentech’s rhGH products. Informed consent was obtained according to the procedures in place at each participating center’s institutional review board. At the time of its closure in June 2010, the database reflected ~ 220,000 GH treatment years in 65,205 children. This database was searched for children with IGHD (peak GH on stimulation testing of <10 ng/mL) or ISS (no identified cause of short stature and peak GH in any test of ≥10 ng/mL) (n = 31,815). Of these, we restricted analysis to those who met the following criteria:

1) Boys ≥4 and <11 years and girls ≥4 and <10 years who were naive to rhGH therapy at enrollment into NCGS.

2) Patients still prepubertal at the end of the second year of therapy defined as testicular volume ≤3 mL in boys and Tanner 1 breast development in girls, in order to eliminate the majority of those who may have started their pubertal growth spurts.

3) Patients with height velocities (HVs) available for calculation of both the first and second years of therapy.

4) Diagnosis of either IGHD (n = 1899) or ISS (n = 1186).

5) Treatment with rhGH administered 6–7 days/week.

Subjects’ HV standard deviation scores (SDS), adjusted for age, sex, and etiology of short stature, were compared not with normal children as a function of age and gender, but with the data of Bakker et al. [5,6] based on HVs during their first and second years of therapy. The Bakker first-year graphs for defining responses to rhGH were derived from NCGS data from 842 females and 2323 males with IGHD and 465 females and 1392 males with ISS [5]. Second-year data were derived from 316 females and 999 males with IGHD and 143 females and 535 males with ISS from the same NCGS database [6] (Additional file 1). Subjects in this study who met the criteria outlined above were then classified by their HV SDS during the first and second years as < −1 (poor responders), −1 to +1 (average responders), or > +1 (best responders).

Mid-parental target height was computed according to Tanner [7] and converted to a SD score. The following characteristics were examined to learn which were predictive of responsiveness to GH: age at baseline, height SDS at baseline, gender, pre-treatment (pre-Rx) HV reported by investigator, body mass index (BMI) SDS at baseline, mother’s height SDS, father’s height SDS, and maximum stimulated GH. Results are presented in tables as means. Wherever the sample size in a single cell is ≤10, data are omitted. *P* values for relationships between prognostic variables and HV SDS were assessed using Pearson correlations.

## Results

Because our subjects span a range of ages from 4 years to 10–11 years, and because the Bakker curves are based on HV SDS, it was felt that presenting the growth data in terms of HV SDS made more sense than trying to express it in cm/year. However, to provide some sense of what our results means in terms of actual growth rates, it should be noted that the first-year HVs reported by Bakker for 8-year-old males with IGHD at the onset of therapy (the approximate average age of the subjects in this report) were HV >11.4 cm/yr for HV SDS >1, whereas those with HV SDS < −1 had HV <5.9 cm/yr. For males with ISS, the HVs for HV SDS >1 were >10.0 cm/yr, whereas those with HV SDS < −1 had HV <5.4 cm/yr. For the second year of treatment, the HVs for HV SDS >1 and −1 for boys aged 9 years (i.e. the ones who were aged 8 years at the beginning of the first year) were 9.6 and 5.7 cm/yr for IGHD and 8.9 and 5.6 cm/yr for ISS.

### Analysis of shift tables

**Figure 1:**
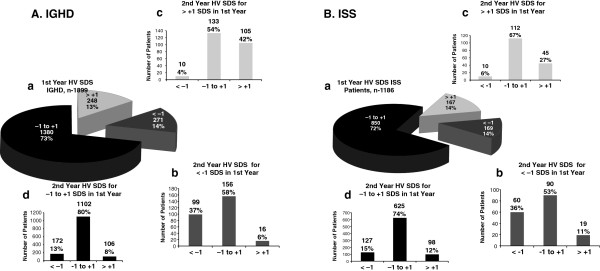
**Distribution of responses based on height velocity standard deviation score (HV SDS) of patients with idiopathic growth hormone deficiency (IGHD) and idiopathic short stature (ISS) in the second year of therapy based on their category of response during the first year.** HV SDS responses to recombinant human growth hormone of patients with IGHD (**A**) and ISS (**B**) in the first year of therapy and subsequent second-year response. The pie segments (a) represent the distribution of patients into the 3 first-year growth velocity groups; < −1 SDS, -1 to +1 SDS, >1 SDS. The three charts, b, c, and d, show the SDS shift in the second year of each of the 3 first-year growth velocity groups.

The number of patients in each HV SDS group and their shifts from year 1 to year 2 are shown in Table 1 and illustrated in Figure 1. Variables assessed are shown in Tables 2, 3, 4 and 5. Each of these shift tables shows the mean results for the variable in question according to each growth response SDS group for the first year in the *last column*. For example in Table 3, the IGHD best first-year growers (HV SDS > +1) had a mean baseline height SDS of −3.2, which is the value in the “Total” column for the “HV SDS > + 1” row. These shift tables also show the total number of patients in each HV SDS group for the second year *in the last row*. To follow a group from their first-year SDS category to their second-year SDS category, begin by looking down the left column of the table to find the growth response category for the first year, and then across the row for the growth response category for the second year. For example, in Table 3 for IGHD, the mean baseline height SDS for patients who were in the SDS > +1 category for the first year and then were in the SDS category between −1 and +1 for the second year is −3.1.

**Table 1:** Numbers of patients in HV SDS groups

**IGHD (n = 1899)**					**ISS (n = 1186)**				
	**Second-year HV SDS**					**Second-year HV SDS**			
**First-Year HV SDS**	< −1	−1 to +1	> +1	Total	**First-Year HV SDS**	< −1	−1 to +1	> +1	Total
< −1	99	156	16	271	< −1	60	90	19	169
−1 to +1	172	1102	106	1380	−1 to +1	127	625	98	850
> +1	10	133	105	248	> +1	10	112	45	167
Total	281	1391	227	1899	Total	197	827	162	1186

*HV*, height velocity; *IGHD*, idiopathic growth hormone deficiency; *ISS*, idiopathic short stature; *SDS*, standard deviation score.

**Table 2:** Mean pre-treatment HV (cm/yr) by first- and second-year HV SDS groups

**IGHD (n = 1044)**					**ISS (n = 680)**				
	**Second-year HV SDS**					**Second-year HV SDS**			
**First-Year HV SDS**	< −1	−1 to +1	> +1	Total	**First-Year HV SDS**	< −1	−1 to +1	> +1	Total
< −1	4.8	4.7		4.7	< −1	4.3	5.4		4.9
−1 to +1	4.4	4.7	4.2	4.6	−1 to +1	4.4	4.6	4.8	4.6
> +1		3.7	4.0	3.8	> +1		3.7	4.5	3.9
Total	4.5	4.5	4.1	4.5	Total	4.4	4.6	4.6	4.6

*HV*, height velocity; *IGHD*, idiopathic growth hormone deficiency; *ISS*, idiopathic short stature; *SDS*, standard deviation score.

**Table 3:** Mean baseline height SDS by HV SDS groups

**IGHD (n = 1899)**					**ISS (n = 1186)**				
	**Second-year HV SDS**					**Second-year HV SDS**			
**First-Year HV SDS**	< −1	−1 to +1	> +1	Total	**First-Year HV SDS**	< −1	−1 to +1	> +1	Total
< −1	−2.9	−2.6	−2.4	−2.7	< −1	−3.1	−2.7	−2.4	−2.8
−1 to +1	−2.8	−2.7	−2.7	−2.7	−1 to +1	−2.8	−2.8	−2.6	−2.8
> +1	−2.8	−3.1	−3.4	−3.2	> +1	−3.3	−2.7	−2.6	−2.7
Total	−2.8	−2.7	−3.0	−2.8	Total	−2.9	−2.8	−2.6	−2.8

*HV*, height velocity; *IGHD*, idiopathic growth hormone deficiency; *ISS*, idiopathic short stature; *SDS*, standard deviation score.

**Table 4:** Mean baseline BMI SDS by HV SDS groups

**IGHD (n = 1891)**					**ISS (n = 1181)**				
	**Second-year HV SDS**					**Second-year HV SDS**			
**First-Year HV SDS**	< −1	−1 to +1	> +1	Total	**First-Year HV SDS**	< −1	−1 to +1	> +1	Total
< −1	−0.5	−0.5	−0.5	−0.5	< −1	−0.8	−0.8	−0.6	−0.8
−1 to +1	−0.5	−0.4	−0.1	−0.4	−1 to +1	−0.6	−0.5	−0.6	−0.5
> +1		0.0	0.1	0.1	> +1	0.7	−0.3	−0.3	−0.2
Total	−0.5	−0.4	0.0	−0.3	Total	−0.6	−0.5	−0.6	−0.5

*BMI*, body mass index; *HV*, height velocity; *IGHD*, idiopathic growth hormone deficiency; *ISS*, idiopathic short stature; *SDS*, standard deviation score.

**Table 5:** Median maximum stimulated GH (ng/mL) by HV SDS groups

**IGHD (n = 1899)**					**ISS (n = 932)**				
	**Second-year HV SDS**					**Second-year HV SDS**			
**First-Year HV SDS**	< −1	−1 to +1	> +1	Total	**First-Year HV SDS**	< −1	−1 to +1	> +1	Total
< −1	6.6	6.7	6.1	6.7	< −1	17.0	14.7	13.8	15.0
−1 to +1	6.3	6.5	4.9	6.4	−1 to +1	14.9	14.2	14.0	14.3
> +1	5.6	4.1	2.0	3.0	> +1		13.9	13.8	14.0
Total	6.4	6.4	3.7	6.1	Total	15.9	14.1	13.9	14.3

*HV*, height velocity; *IGHD*, idiopathic growth hormone deficiency; *ISS*, idiopathic short stature; *SDS*, standard deviation score.

### Patients with IGHD

#### Characteristics

There were 1507 boys and 392 girls (21%) included in the analyses. Mean age at start of therapy (7.3 ± 1.9 years) was similar in all three HV groups and is not shown. Also not shown in the tables was the mean GH dose (0.3 ± 0.05 mg/kg/week), which was nearly identical in all three groups. The greatest year 1 HV SDS was associated with lower mean pre-Rx HV (3.8 cm/yr vs. 4.7 and 4.6 cm/yr for the poor and average responders, respectively, *P* <0.0001; Table 2). However, the correlation of the pre-treatment HV with the first-year Bakker HV SDS was not large (*r* = 0.16). The best responders also had a lower mean baseline height SDS (−3.2 vs. − 2.7 and −2.7 for the poor and average responders, *P* <0.0001; Table 3), greater mean BMI SDS (+0.1 vs. −0.5 and −0.4, *P* < 0.0001, Table 4), and lower median maximum stimulated GH (3.8 vs. 6.7 and 6.4 ng/mL, *P* <0.0001, Table 5). In every case, the difference between the best and average responder groups was much greater than between the average and poor responders. The effect of parental height is shown in Table 6. The height SDS deficit at baseline is equal to the mid-parental target height SDS minus the height SDS of the patient at baseline. For the first year of treatment, the deficit was 3.1 in the IGHD best responders and 2.2 in the poor responders. For the second year, the deficit was 2.8 in the best responders and 2.3 in the poor responders. The bigger the difference between the mid-parental target height SDS and the patient’s baseline height SDS, the greater the HV was likely to be in both the first and second year of GH treatment (*P* <0.0001 and *P* <0.0001).

**Table 6:** Mean mid-parental target height SDS minus baseline height SDS by HV SDS groups

**IGHD (n = 1664)**					**ISS (n = 1001)**				
	**Second-year HV SDS**					**Second-year HV SDS**			
**First-Year HV SDS**	< −1	−1 to +1	> +1	Total	**First-Year HV SDS**	< −1	−1 to +1	> +1	Total
< −1	2.4	2.0	2.0	2.2	< −1	2.5	2.1	1.8	2.2
−1 to +1	2.3	2.2	2.4	2.2	−1 to +1	2.2	2.2	2.2	2.2
> +1		2.9	3.3	3.1	> +1		2.5	2.4	2.5
Total	2.3	2.3	2.8	2.3	Total	2.3	2.2	2.2	2.2

*HV*, height velocity; *IGHD*, idiopathic growth hormone deficiency; *ISS*, idiopathic short stature; *SDS*, standard deviation score.

To address the possibility that our results might have been biased by preferential drop-out in the second year of patients who had a poor response to GH treatment in the first year, we looked at a separate subset of patients for whom we had HV data for the first year but no second year data (*N* = 352) either because of early drop-out or because there were no visits within the time frame needed for the second-year time point. We found a regression coefficient of −0.290 for the Bakker HV SDS (*P* <0.0001), suggesting that those IGHD subjects with poorer growth during the first year of growth hormone therapy were more likely to drop out than those with better first-year growth.

As shown in Table 7, we defined subgroups of patients with IGHD who had all of the characteristics we identified as predictive of excellent response (n = 28) vs. patients who would have been predicted to respond poorly (n = 29). The patients predicted to be “super-responders” did indeed have a very strong first-year response to GH (increase in HV from 1.9 to 13.1 cm/yr [compared with an average first-year HV of 11.4 cm/yr for the best responder group based on the Bakker curves for 8-year-old males]). The group with poor predictors still responded to GH, albeit with a lesser response (increase from 6.2 to 8.6 cm/yr). The poor response group based on the Bakker curves had an average first-year HV of 6.7 cm/yr, so we were much less successful in using our predictors to define a group of very poor responders than to define a group of super-responders. When we looked at growth of the same group of subjects during their second year of GH therapy, the difference in HV and HV SDS between the predicted best and worst responders was much smaller, confirming that first-year growth may not be very predictive of growth in subsequent years.

**Table 7:** Comparison of first-year growth for patients with IGHD who had all the characteristics of the best responders with those predicted to be poor responders

**Variable**	**Predicted best first-year HV SDS criterion n = 28**	**Predicted poor first-year HV SDS criterion n = 29**
Baseline height SDS	< −3	> −2.5
Baseline BMI SDS	> −0.5	< −1
Pre-treatment HV (cm/yr)	< 3.5	> 4
Baseline height SDS minus mid-parental target height SDS	> 3	< 2
Maximum stimulated GH (ng/mL)	≤ 4	≥ 7
Female (n)/male (n)	5/23	7/22
	Mean (SD)	Mean (SD)
Baseline age (yr)	6.5 (1.8)	6.8 (2.0)
Baseline height SDS	−4.1 (0.7)	−1.9 (0.3)
Baseline BMI SDS	0.5 (0.8)	−1.5 (0.5)
Pre-treatment HV (cm/yr)	1.9 (1.0)	6.2 (1.9)
Baseline height SDS minus mid-parental target height SDS	4.2 (0.7)	1.4 (0.5)
Maximum stimulated GH (ng/mL)	1.9 (1.0)	8.6 (0.8)
First-year HV (cm/yr)	13.1 (2.7)	9.1 (2.3)
First-year Bakker HV SDS	1.21 (1.3)	−0.51 (0.9)
Second-year HV (cm/yr)	8.5 (2.0)	7.3 (1.4)
Second-year Bakker HV SDS	0.33 (1.3)	−0.37 (0.8)

*BMI*, body mass index; *HV*, height velocity; *GH*, growth hormone; *SDS*, standard deviation score.

#### Shifts

During year 2, 172 of 271 (73%) poor responders moved to either the average (n = 156) or best responder groups (n = 16), with 99 remaining poor responders. Four percent of the best responders moved to the poor responder group (Table 1 and Figure 1, panel A). Year 2 HV SDS was associated with having a greater difference between baseline height SDS and mid-parental target height SDS, greater BMI SDS, and lower maximum stimulated GH. Twenty percent of the variance of HV SDS in year 2 was predicted by HV SDS in year 1 (*r* = 0.45, *P* <0.0001), using HV SDS as a continuous variable.

### Patients with ISS

#### Characteristics

There were 928 boys and 258 girls (22%) included in the analyses. Numbers of patients in each HV SDS category and their shift from year 1 to year 2 are shown in Table 1 and Figure 1, panel B. Variables assessed are shown in Tables 2, 3, 4 and 5. Mean age at start of therapy (7.7 ± 1.9 years) was similar in the 3 groups and similar to the IGHD group. Not shown in the tables but also assessed was GH dose (0.31 ± 0.06 mg/kg/wk), which was nearly identical in all groups and almost the same as for IGHD. Greater year 1 HV SDS was associated with lower pre-Rx HV (3.9 cm/yr vs. 4.9 and 4.6 cm/yr for the average and poor responders, *P* <0.0001, Table 2). However, the correlation of pre-treatment HV with the first-year Bakker HV SDS was not large (*r* = 0.15). Greater year 1 HV SDS was not associated with baseline height SDS (Table 3) in contrast to the IGHD group. Greater year 1 HV SDS was associated with greater mean BMI SDS (−0.2 vs. −0.8 and −0.5, *P* <0.0001, Table 4); unlike in the IGHD group, it was not associated with stimulated GH, which by definition was >10 ng/mL in all patients (Table 5). There were no significant differences in responses between boys and girls. The greater the difference between the mid-parental target height SDS and the patient’s baseline height SDS, the greater the HV was likely to be during the first year of GH treatment (*P* <0.022) but not during the second year (*P* = 0.090) (Table 6). Only 7% of the variance of HV SDS in year 2 was predicted by HV SDS in year 1 (*r* = 0.27, *P* <0.0001). The difference in mean HV SDS between ISS patients with both first- and second-year HV and patients with first- but not second-year HV was not significant (*P* = 0.21).

#### Shifts

During year 2, 109 of 169 (64%) poor responders moved to the average responder (n = 90) or best responder groups (n = 19), with only 60 (36%) remaining poor responders (Table 1 and Figure 1, panel B). Six percent of the best responders moved to the poor responder group. Having the best HV SDS in year 2 was not notably related to any of the baseline characteristics examined in Tables 2, 3, 4 and 5. Only 7% of the variance of HV SDS in year 2 was predicted by HV SDS in year 1 (*r* = 0.27, *P* <0.0001), using HV SDS as a continuous variable.

## Discussion

Previous studies looking at patients with various etiologies of short stature either at single institutions or from large databases such as the Kabi International Growth Study (KIGS) or the ANSWER Program have attempted to define pre-Rx factors that best predict short-term GH responsiveness [1-4]. Goals of these studies have been to inform the selection of patients most likely to benefit from GH therapy, to predict the magnitude of the increase in HV during therapy, and to help pick the optimal dose of GH to produce the desired increase in HV. Younger age has been found to predict better responsiveness to GH, as well as lower peak GH levels in response to provocative testing, taller parents, and a higher BMI SD score. In patients with IGHD, these predictors make clinically relevant “sense” since they define the characteristics of this group, and previous studies have shown that the most severely GH-deficient children tend to be the most responsive to GH [1,3,4].

An earlier report using the NCGS database showed that the increase in height SDS during the first year of GH therapy was significantly greater in patients with peak GH < 3 ng/mL (+1.14) than in those with peak GH of 3 to 7 ng/mL (+0.81) and 7 to 10 ng/mL (+0.72) [8]. In addition, children with severe GH deficiency tend to have increased body fat stores, as noted in a study that found a high correlation between baseline leptin and first-year change in height SDS (*r* = 0.49; *P* <0.0001) in a sample of 150 Swedish children with a range of peak GH values [9]. Thus it is not surprising that the best responders in our IGHD group have a significantly higher BMI SDS than the average responders, although the difference in mean BMI SDS between the average and poor responders was relatively small. In the ISS group, BMI was also positively associated with response. Parental height clearly also contributes to responsiveness, as short children of average or tall parents have greater genetic potential and usually have a greater height deficit to recoup during the first 2 years of therapy. For IGHD patients, the bigger the difference between the mid-parental target height SDS and the patient’s baseline height SDS, the greater the HV was likely to be during both the first and second years of GH treatment, but for ISS patients, the effect of this difference on HV was much smaller during the first year and non-significant for the second year.

While our data confirm some of the predictors already known, our approach to defining GH responsiveness in patients with IGHD and ISS differs from other approaches in several respects. We were able to define the best and the poorest responders not based on arbitrary criteria, such as a certain increase in HV or in height SDS, factors that depend to some extent on the age of the patients, but based on age, sex, and diagnosis-specific HV curves for both the first and second years of GH therapy from large numbers of similarly treated patients. These curves, developed by Bakker et al. [5,6] were derived from a large sample of GH-treated patients with IGHD and ISS contained within the same NCGS database as was used for our analyses. We also wanted to examine both the best and the poorest responders using the same baseline criteria to see whether the factors associated with poor response deviated from those found in the average responders to the same degree but in the opposite direction as the best responders. The fact that there was a much greater separation between the best and average responders compared with the average and poor responders suggests that the best responders make up a more distinct subset of patients than do the poor responders. Poor response to GH may stem from factors we have not assessed, such as undefined genetic differences in skeletal responsiveness and poor compliance with treatment. While we do not have any data on compliance, such issues with GH treatment are not uncommon [10,11]. Age and dose were eliminated as predictors of response for both IGHD and ISS in our analysis because they were similar between the comparison groups. Of the pre-Rx variables that differed between the patients, we found that the best responders in both the IGHD and ISS groups had lower pre-Rx HVs and higher BMI, with differences seen only during the first year of therapy; second-year responsiveness to GH was not related to pre-Rx HV. Lower peak stimulated GH and taller parents predicted second-year responsiveness in the patients with IGHD; there were no strong predictors of second-year responsiveness in the ISS group.

For comparison, in an analysis of 169 Swedish prepubertal children with a mean age of 8.3 years and a wide range of stimulated GH levels, all treated with 0.1 U/kg/day, Kristrom et al. reported that the maximum GH after arginine-insulin, age at start of treatment, mid-parental height SDS, pre-Rx height SDS and HV, and the difference between pre-Rx height SDS and mid-parental height SDS (diff SDS) all showed significant correlations (*r* = 0.22 to 0.43) with the increase in height SDS over 2 years of therapy [1]. By stepwise linear regression, diff SDS and log GH_max_ were the strongest predictors. Using the KIGS database for patients with only IGHD, Geffner and Dunger [12] reported that degree of GH deficiency, age, height – mid-parental height SDS, and weight SDS (which correlated strongly with height SDS) were, in that order, the variables most predictive of HV during the first year of therapy. For children with ISS, Ranke et al. [13] reported that by multivariate linear regression, the four variables associated with first-year response to GH were age, GH dose, height – mid-parental height SDS, and weight SDS. In both examples, if you eliminate age and dose, the degree of GH deficiency remains the most consistent variable.

For IGHD, it is clear that the best response in the first and second years was seen in the patients with the greatest difference between starting height and the target height. More recently, Lee et al., using the ANSWER study database involving 698 children with IGHD, found that the change in HV at 4 months, baseline age, baseline height SDS, and baseline BMI SDS were, in that order, the best predictors of the increase in height SDS at 1 and 2 years after starting GH; they did not report predictors of response for their smaller group of 123 children with ISS [3]. We found more predictors of excellent response to GH therapy in the IGHD group than in the ISS group, and several of these predictors for IGHD but not for ISS had significant effects into the second year.

One unique aspect of this study was our ability to see how the classification of IGHD and ISS responses to rhGH, which were found during the first year of therapy, changed when these groups were followed into the second year. One might have predicted that the best and poorest responders during the first year would largely remain in the same categories during the second year, but this was not the case. While a large proportion (42%) of patients with IGHD who were best responders in the first year remained in this group during the second year, and relatively few (4%) became poor responders, a greater proportion (58%) of the IGHD first-year poor responders became average responders during the second year, with only 37% remaining poor responders during the second year. In the ISS group, the comparable second-year numbers were 53% moving from poor to average responders and 36% remaining poor responders. A potential weakness of the analysis is that our results may have been biased by the possibility that some children who responded poorly during year 1 may have been taken off treatment by either the physician or by the family; they would not show up in our analysis because we only looked at patients completing 2 years of therapy. Our drop out analysis confirms that bias since those who did drop out tended to have a somewhat poorer height velocity, albeit only about 5% on average less than those who remained in the study for 2 years. Nevertheless, most of those who grew poorly in the first year but who continued treatment grew satisfactorily or well during the second year.

There are important implications of these findings. First, as suggested above, poor responders may be more similar to average responders than to best responders. Second, as Tanner et al. demonstrated some 46 years ago [14], in general, the correlation of height gain in prepubertal children from year to year is only about 0.3 and this may underlie growth responsiveness to hormonal treatment as well. Compliance may also be a factor as could changes in the psychosocial environment [15]. Thus, these findings suggest that a suboptimal first-year growth response does not always predict a continued poor response in the second year. The treating physician may consider continuing therapy into the second year, with appropriate attention to factors such as compliance and psychosocial factors, and the knowledge that there may be episodic changes in HV that are inherent to the growth process and not obscured by GH treatment.

## Conclusion

In an analysis based on comparing patients with IGHD of the same age and rhGH dose, the characteristics contributing to the best first- and second-year responses were those that describe the patients with classical IGHD: lower pre-Rx HV, lower baseline height SDS, higher BMI SDS, taller parents, and lower maximum stimulated GH. For patients with ISS, few characteristics stood out. During the first year, lower pre-Rx HV and higher BMI SDS predicted better growth. Year 1 HV SDS, greater baseline height SDS and lower stimulated GH predicted second-year HV SDS. For nearly all criteria examined, the difference between the best and average responders was much greater than the difference between average and poor responders, suggesting that factors we were not able to assess (e.g. inherent biologic and genetic variation in growth, compliance, and social factors) may contribute more to their suboptimal response to therapy. For both IGHD and ISS, HV SDS in the first year was not a strong predictor of HV SDS in the second year, indicating that the use of the first-year HV alone to make a decision regarding GH continuation is not supported by these data.

## Abbreviations

BMI: Body mass index; GH: Growth hormone; HV: Height velocity; IGHD: Idiopathic growth hormone deficiency; ISS: Idiopathic short stature; KIGS: Kabi International Growth Study; NCGS: National Cooperative Growth Study; rhGH: Recombinant human growth hormone; Rx: Treatment; SDS: Standard deviation score.

## Competing interests

PBK and DIS have no conflicts to disclose, JWF is a biostatistician consultant for Genentech, JJ is a Genentech employee, BL is a medical affairs consultant for Genentech.

## Authors’ contributions

PBK developed the original concept of looking at the characteristics of the best responders to GH in the NCGC database, suggested the variables to be assessed and wrote the introduction and much of the discussion. DIS contributed the concept of looking at the characteristics of the poor responders and combining it with the best responders, and reviewed the early and final drafts of the manuscript. JJ participated in the study design and statistical analysis, developed graphic displays, and contributed to the statistical methods section along with JWF. JWF was the statistician that performed the analyses for the manuscript after consultation with the other authors; contributed to the text of the manuscript and reviewed all of the contributions to the manuscript from the other authors.; and read and approved the final manuscript. BL was responsible for determining the focus and type of data to be extracted and analyzed from the NCGS database; contributed to the design and content presentation of the data; reviewed the contribution of the statistician and organized the data presentation; and contributed to the review of the final manuscript. All authors read and approved the final manuscript.

## Supplementary Material

Additional file 1 Height velocity in short children during second and third year of growth hormone treatment. Click here for file
