# Supplementary material for: Characteristics of children with the best and poorest first- and second-year growth during rhGH therapy: data from 25 years of the Genentech national cooperative growth study (NCGS)
Source: Int J Pediatr Endocrinol. 2013 May 1;2013(1):9. doi: 10.1186/1687-9856-2013-9 (PMC3660178; doi:10.1186/1687-9856-2013-9)
Supplement: Additional file 1 — Height velocity in short children during second and third year of growth hormone treatment. [file 1687-9856-2013-9-S1.pdf]

Height Velocity in Short Children During Second and Third Years of Growth Hormone (GH) Treatment: Data From the National Cooperative Growth Study (NCGS)

Bert Bakker,[1] Jim Frane,[2] Henry Anhalt,[3] Barbara Lippe,[1] Ron G. Rosenfeld[4]

[1]Genentech, Inc., South San Francisco, CA; [2]Consultant, Santa Monica, CA; [3]Animas Corporation, West Chester, PA; [4]The Lucile Packard Foundation for Children's Health, Palo Alto, CA

INTRODUCTION

- Although GH has been used to treat short stature in GHD and other conditions for over 40 years, criteria for satisfactorily defining targets for GH responsiveness for multiple years of treatment have never been developed.
- The range of GH response is large; differences can be attributed to diagnosis, age, GH dose, parental height, compliance, intercurrent illness, other (endocrine) therapies, and still poorly defined molecular and biochemical factors.
- We recently published HV targets from NCGS for first-year GH responses in short children.[1] Data from 8442 prepubertal boys and 4328 girls with IGHD, OGHD, ISS, or TS were used.
- To further develop reference data to aid clinicians in defining GH responsiveness, we used data from Genentech's NCGS to construct plots of height velocity during the first 3 years of treatment with standard QD GH doses in prepubertal patients, aged 2-14, with IGHD, OGHD, ISS, and TS.

METHODS

- The NCGS was initiated in 1985 following the withdrawal of pituitary-derived GH because of Creutzfeldt-Jakob disease and in response to a request by the US Food and Drug Administration to monitor the efficacy and safety of recombinant human growth hormone (rhGH). Since that time, the NCGS registry has grown to become the largest and longest-standing repository of GH-related data in North America, containing demographic and outcomes data from more than 55,000 children with growth-related disorders.
- Using data from the NCGS, we studied second- and third-year growth response to GH expressed as HV in 4 common growth disorders. We selected subjects at least 2 years of age whose weekly GH doses were between 0.27 and 0.33 mg/kg, with injection frequencies around 6 or 7 a week.
- Among these subjects, we further selected subjects who had been identified by the NCGS investigators as having OGHD, ISS, or TS, together with subjects identified as having confirmed IGHD.
- For each gender and etiology, mean ± 1 SD first-, second-, and third-year HV on GH were plotted vs subject age for subjects who remained prepubertal at the end of the treatment year in question. A smooth curve was fit as a function of age through the growth rates of individual subjects using the nonparametric regression Loess function from SAS®, which computes a smooth curve defining the mean growth at each age.[2]
- The absolute values of the residuals from this first fit were computed. Next, these residuals were fit vs age, again, using Loess SAS function. Last, the SD was taken as the square root of π/2 times this second fit (using the relationship between the mean of a half-normal distribution with the SD of the corresponding normal distribution).
- As an example of the fitting procedure, the resulting raw data for the first year of GH treatment with fitted curves are illustrated in Figure 1 for boys with IGHD. The curves for the mean ± 1 SD are depicted (see also reference 1).

Figure 1: Mean and Mean ± 1 SD for Height Velocity During First Year of QD GH Treatment (0.27 to 0.33 mg/kg/wk) for Naive Prepubertal IGHD Males

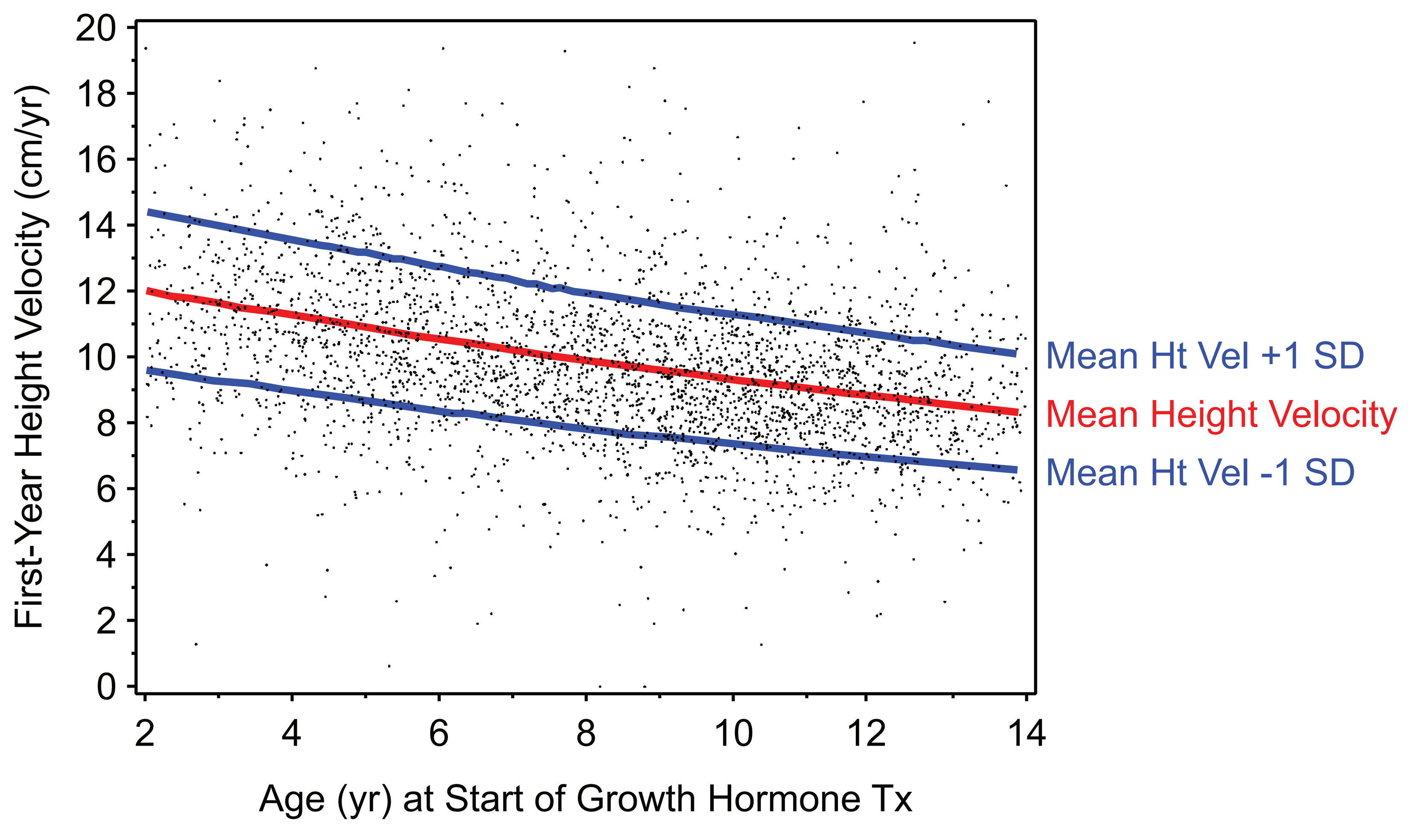

- Sample sizes for fitting curves were:

Group	Females 1st year	Females 2nd year	Females 3rd year	Males 1st year	Males 2nd year	Males 3rd year
IGHD	574	316	224	1669	999	699
OGHD	239	144	135	411	269	224
ISS	338	143	100	1064	535	295
TS	498	301	195	–	–	–

RESULTS

- The 21 plots in Figure 2 show the mean ± 1 SD for HV for females and males with IGHD, OGHD, ISS, and TS.

Figure 2: Mean ± 1 SD Height Velocity During the First 3 Years of QD GH Treatment (0.27 to 0.33 mg/kg/wk) for Naive Prepubertal Patients

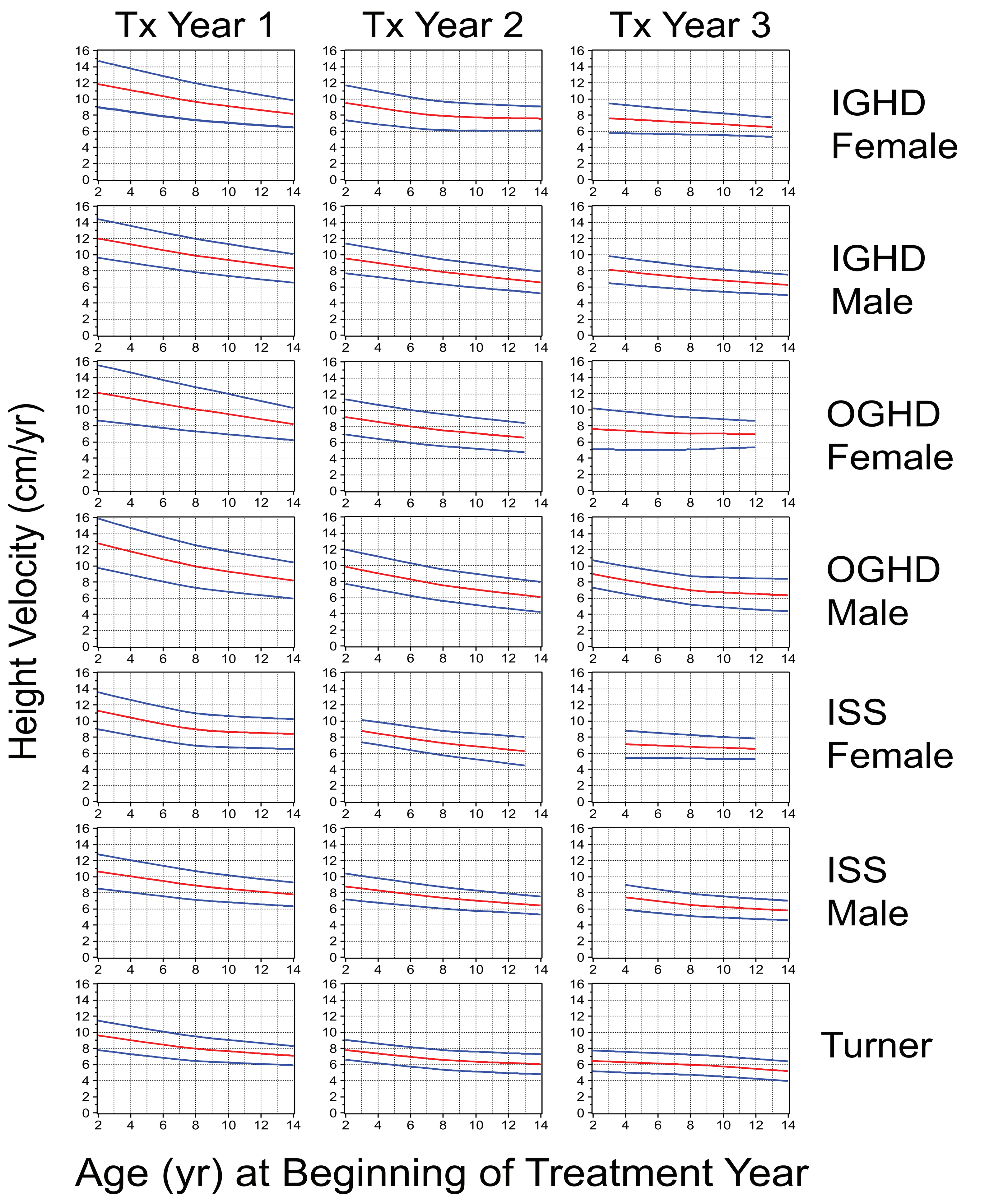

- As was demonstrated before (in the first-year HV plots), the mean HV varies with age and declines as age increases.[1] The SD of the HV varies only somewhat with age.
- The HV data for the second and third year also demonstrate the expected decreases in HV relative to year of GH treatment.

DISCUSSION

- These NCGS-based HV plots for the first, second, and third year of GH treatment show that the mean height velocities for IGHD, OGHD, ISS, and TS vary with age and GH treatment year, and also that the SD varies somewhat with age. The HV plots should be viewed as conservative because of unknown diagnostic issues and undocumented compliance in subjects in the NCGS.
- Nevertheless, these HV plots provide a good foundation for evaluating the first year (previously published[1] and repeated in this poster), and *second* and *third* year (new in this poster) of growth responses in children receiving GH.
- While the data for the second and third year demonstrate the expected decreases in HV relative to year (of GH treatment) and age, the actual means ± 1 SD quantify the responses for each etiology in a manner not previously illustrated.
- The optimal HV target should be individualized, in each year, for degree of initial catch-up growth obtained, goals for subsequent catch-up, and factors such as estimated time before puberty.
- These HV plots can serve as a practical tool for clinicians to use when comparing the first-, second-, and third-year HV of specific patients with the HV of a large population in the NCGS database.
- If the HV in any of these years of GH treatment is *below* –1 SD of the calculated HV from the database, clinicians should reconsider whether:
  a) The diagnosis is correct
  b) There is comorbidity, such as undiagnosed underlying illness
  c) Compliance is adequate
  d) The GH dose needs adjustment
  e) Undetermined factors are affecting the clinical response to GH, such as defects of GH and/or IGF sensitivity

CONCLUSION

The use of age at baseline-, gender- and etiology-specific first-, second-, and third-year HV plots offer the clinician a benchmark against which to assess the progress of an individual patient and can contribute to evidence-based decision making to maximize the efficacy of GH treatment.

REFERENCES

1. Bakker B, Frane J, Anhalt H, Lippe B, Rosenfeld RG, 2008. Height velocity targets from the National Cooperative Growth Study for first-year growth hormone response in short children. J Clin Endocrinol Metab 93:352-357.
2. Cleveland W, Grosse E, 1991. Computational methods for local regression. Stat Comput 1:47-62.
